# Alleged Misinterpretation of PET-CT in Esophageal Carcinoma Staging: A Medico-Legal Case Report

**DOI:** 10.7759/cureus.101664

**Published:** 2026-01-16

**Authors:** Felipe R De Queiroz, Natalia V Jordão, Daniel T Machado

**Affiliations:** 1 Judicial Medical Expert Examinations Department, Judiciary of Brazil, Court of Justice of the State of Minas Gerais (TJMG), Belo Horizonte, BRA; 2 Emergency Department, Cooperativa de Trabalho Médico de Belo Horizonte (Unimed-BH), Belo Horizonte, BRA

**Keywords:** cancer staging, diagnostic ct imaging, esophageal squamous cell carcinoma, medical malpractice defense, medico-legal case, pet-ct scan

## Abstract

Diagnostic imaging is pivotal in the staging of esophageal carcinoma, yet interpretation of metabolic findings can be challenging because positron emission tomography/computed tomography (PET/CT) is highly sensitive but may lack specificity, sometimes limiting differentiation between primary and metastatic lesions without histopathological correlation. We report a medico-legal case involving an alleged misdiagnosis in which a metastatic lymph node was perceived as a synchronous pancreatic malignancy, purportedly delaying treatment. A 65-year-old female presented with upper abdominal discomfort and was diagnosed with esophageal squamous cell carcinoma via endoscopy. Subsequent staging with 18F-FDG PET/CT revealed a hypermetabolic lesion in the pancreatic body, characterized as neoplastic, with a recommendation for clinical correlation. The patient interpreted this finding as a second primary cancer (pancreatic), resulting in significant distress. An endoscopic ultrasound-guided biopsy subsequently confirmed that the lesion represented a metastatic lymph node of esophageal origin. The patient underwent palliative chemotherapy but died from disease progression 14 months after diagnosis. A legal claim alleged that the diagnostic investigation of the presumed "pancreatic mass" delayed life-saving treatment. This case highlights the limitations of PET/CT specificity and the critical role of tissue diagnosis in oncologic staging; medico-legal analysis indicated that the imaging report met the standard of care by identifying the abnormality and recommending biopsy. Expert review concluded that the timeline of care was appropriate for complex staging and that the outcome was driven by the aggressive biology of stage IV esophageal cancer rather than diagnostic delay, underscoring the need to manage patient expectations regarding imaging uncertainty and reaffirming that adherence to diagnostic algorithms, including confirmatory biopsy, constitutes the standard of care even when it may be perceived as causing delays.

## Introduction

Esophageal cancer remains one of the most challenging malignancies to treat globally, characterized by aggressive biological behavior and a propensity for early lymphatic and hematogenous dissemination [1]. It is the seventh most common cancer and the sixth leading cause of cancer death worldwide [1]. The majority of patients present with advanced disease, making accurate initial staging the most critical factor in determining prognosis and selecting the appropriate therapeutic strategy. The staging process typically involves a multimodal approach, including upper gastrointestinal endoscopy, endoscopic ultrasound (EUS), computed tomography (CT), and, increasingly, 18F-fluorodeoxyglucose positron emission tomography/computed tomography (FDG-PET/CT) [1].

FDG-PET/CT has revolutionized the management of esophageal carcinoma by offering functional metabolic information that complements anatomical imaging. It is particularly valued for its high sensitivity (ranging from 71% to 92%) in detecting distant metastases that may be occult on conventional CT scans [1]. The detection of such metastases is pivotal because it upstages the disease to Stage IV (M1), thereby contraindicating curative surgical resection and shifting the management focus to systemic palliative therapy. However, the utility of PET/CT is tempered by its inherent limitations regarding specificity. FDG is a glucose analog that accumulates in tissues with high glycolytic activity. While this is a hallmark of malignant cells, it is not exclusive to them; inflammatory processes, infectious conditions, and benign physiological uptake can also demonstrate significant FDG avidity, leading to potential false-positive interpretations [2].

Furthermore, a specific diagnostic dilemma arises when distinguishing between primary synchronous malignancies and metastatic lesions. In patients with esophageal cancer, the identification of a hypermetabolic mass in an adjacent organ, such as the pancreas, presents a differential diagnosis that includes direct tumor invasion, metastatic lymphadenopathy, or a synchronous second primary pancreatic adenocarcinoma. Differentiating these entities based solely on metabolic imaging is often impossible, as both primary and secondary malignancies exhibit high standardized uptake values (SUVs) [2,3]. Consequently, clinical guidelines universally mandate histopathological confirmation of PET-positive findings before denying a patient potentially curative therapy or initiating toxic palliative regimens [3,4].

The gap between the technical reality of diagnostic uncertainty and the patient's expectation of absolute precision can lead to significant distress and, in some instances, litigation. Patients may perceive the identification of a "new mass" not as a step in the staging process, but as a definitive diagnosis of a second, often fatal, condition. When further testing is required to clarify these findings, the resulting time interval can be viewed by patients and their families as an unacceptable delay in treatment, potentially fueling malpractice claims if the clinical outcome is poor [5].

This article presents a comprehensive medico-legal case report of a 65-year-old female with esophageal squamous cell carcinoma. The case centers on an 18F-FDG PET/CT scan interpretation that identified a hypermetabolic lesion in the pancreatic region. The ambiguity of this finding - whether it represented a metastatic lymph node or a synchronous pancreatic cancer - necessitated additional invasive testing, allegedly delaying the initiation of chemotherapy. Following the patient's death from disease progression, a lawsuit was filed claiming that the "misdiagnosis" of pancreatic cancer and the subsequent diagnostic delay contributed to the fatal outcome. We provide a detailed account of the clinical course, followed by an extensive discussion of the medical and legal principles regarding standard of care, diagnostic specificity, and the establishment of causation in oncology malpractice litigation.

## Case presentation

Patient information and social history

The patient was a 65-year-old female, retired, residing in an urban setting with good access to healthcare facilities. She had no significant past medical history, specifically no history of prior malignancies, diabetes, or autoimmune disorders. Her social history was unremarkable; she was a non-smoker and did not consume alcohol, which are typically major risk factors for squamous cell carcinoma of the esophagus. Her functional status at presentation was preserved (ECOG (Eastern Cooperative Oncology Group) Performance Status 0-1), although she reported recent fatigue associated with her symptoms.

Clinical presentation

In October 2022, the patient presented to her primary care physician with a chief complaint of new-onset, persistent upper abdominal discomfort centered in the epigastrium. She described the sensation as a vague, gnawing pain unrelated to meals. She also reported mild digestive symptoms, including early satiety. Notably, she did not report classic dysphagia (difficulty swallowing) or significant unintentional weight loss at the initial consultation, which often delays the suspicion of esophageal malignancy. Physical examination of the abdomen was benign, with no palpable masses or organomegaly. Routine laboratory blood work was largely within normal limits.

Diagnostic assessment and imaging findings

Given the persistence of epigastric symptoms despite symptomatic management, the patient was referred for an upper gastrointestinal endoscopy.

Endoscopic Findings (October 13, 2022)

**Figure 1:**
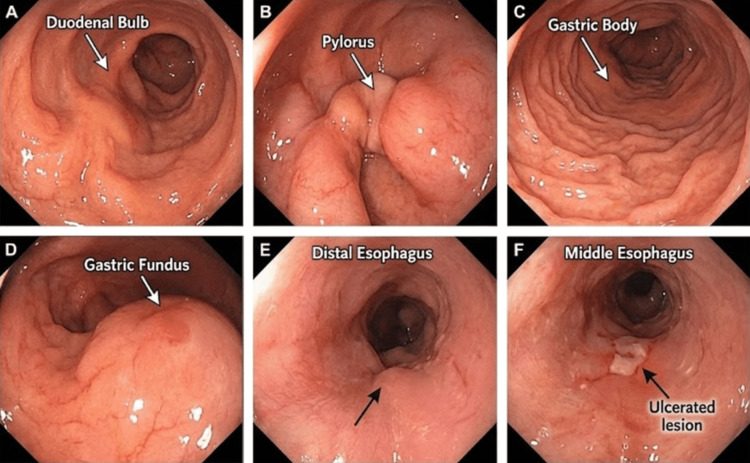
Endoscopic views of the upper gastrointestinal tract A. Duodenal bulb: view showing the first part of the duodenum B. Pylorus: view of the pyloric sphincter connecting the stomach to the duodenum C. Gastric body: view showing the rugal folds within the body of the stomach D. Gastric fundus: view of the upper part of the stomach E. Distal esophagus: view showing the distal esophagus near the gastroesophageal junction F. Middle esophagus: view of the middle esophagus showing a white, ulcerated lesion (arrow)

The endoscopic examination revealed a significant abnormality in the distal esophagus, located approximately 31 cm from the dental arch. The endoscopist described an ulcerated lesion measuring approximately 1 cm in diameter. The lesion exhibited friable edges and spontaneous bleeding upon contact and was covered by a fibrinous exudate (Figure 1). The endoscopic impression was highly suspicious for a malignant process. Biopsies were taken from the lesion, as well as random biopsies from the gastric antrum and body to rule out* Helicobacter pylori* infection or synchronous gastric pathology.

Histopathology

The pathology report, finalized on October 18, 2022, confirmed the diagnosis. The esophageal biopsies demonstrated invasive squamous cell carcinoma, moderately differentiated. The tumor cells showed keratinization and invasion into the subepithelial tissue. Gastric biopsies revealed only mild chronic gastritis, with no evidence of malignancy or *H. pylori* infection.

Computed Tomography (October 20, 2022)

**Figure 2:**
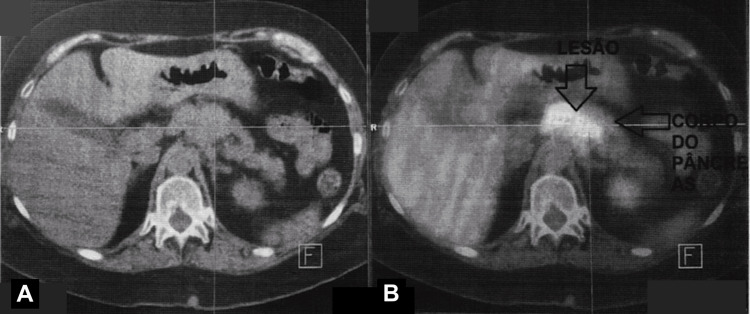
Axial imaging of the gastroesophageal junction A. Axial CT image: contrast-enhanced scan showing a heterogeneous soft-tissue mass at the gastroesophageal junction B. Fused PET/CT image: metabolic fusion image highlighting the hypermetabolic lesion (upper arrow, "LESÃO") and its proximity to the pancreatic body (lower arrow, "CORPO DO PÂNCREAS"), demonstrating loss of distinct fat planes

To assess local tumor extent and screen for metastases, a contrast-enhanced CT scan of the chest, abdomen, and pelvis was performed. The scan revealed a heterogeneous soft-tissue mass located at the gastroesophageal junction. The mass measured approximately 4-5 cm in its largest dimension, significantly larger than the surface lesion seen on endoscopy, suggesting submucosal or adventitial spread. Crucially, the radiologist noted that the mass encased the left gastric artery and abutted the posterior aspect of the pancreatic neck. The report emphasized that there was "no clear cleavage plane" between the tumor and the pancreas, raising the possibility of direct invasion (Figure 2). Additionally, enlarged retroperitoneal lymph nodes were identified in the celiac axis. No pulmonary or hepatic metastases were visualized. The working diagnosis was locally advanced esophageal carcinoma (cT3-4 N+ M0).

18F-FDG PET/CT (November 4, 2022)

**Figure 3:**
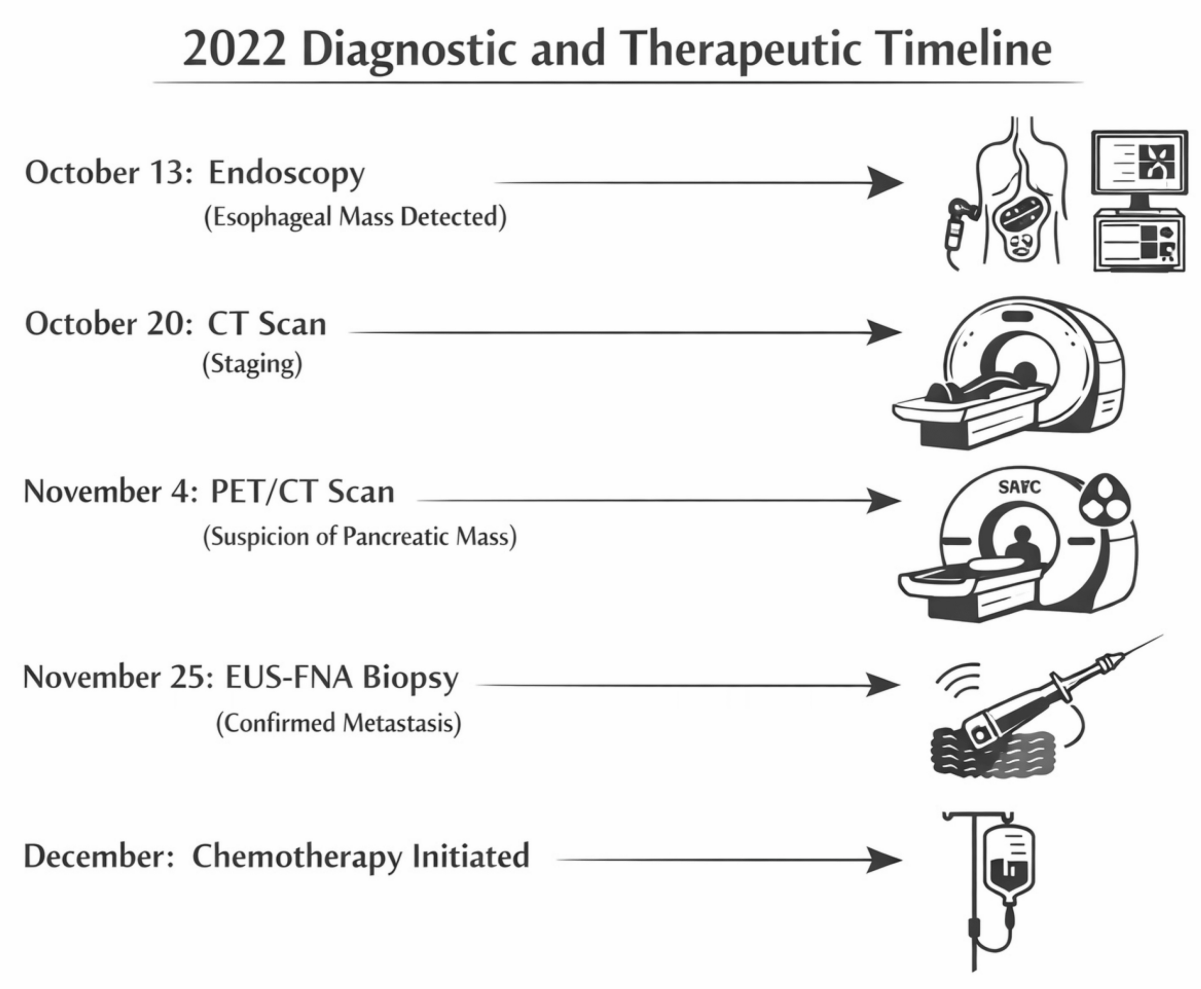
Timeline of diagnostic and therapeutic interventions A timeline illustrating the dates of the endoscopy (October 13), CT scan (October 20), PET/CT scan (November 4), EUS-FNA biopsy (November 25), and start of chemotherapy (December 2022) EUS-FNA: endoscopic ultrasound with fine-needle aspiration

The oncology team recommended a PET/CT scan to complete the staging workup, specifically to rule out occult distant metastases that would contraindicate radical surgery (esophagectomy). The patient underwent the scan at a private imaging center. The PET/CT demonstrated intense hypermetabolism at the primary tumor site in the distal esophagus. However, the most significant finding was a distinct, intensely hypermetabolic lesion in the region of the pancreatic body. This lesion measured 3.4 × 3.6 cm and exhibited an SUVmax of approximately 13.0, indicating very high glucose metabolism. The radiology report described this as a "hypermetabolic lesion in the pancreatic body, consistent with a process of neoplastic nature." The report provided a differential diagnosis: it could represent a large metastatic lymph node in the celiac/pancreatic region or, less likely, a synchronous primary pancreatic malignancy. The radiologists explicitly advised correlation with clinical data and histopathological confirmation. They did not definitively diagnose pancreatic cancer, but the description of a "neoplastic lesion in the pancreas" was alarming [2]. The overall sequence of diagnostic and therapeutic steps is summarized in the timeline (Figure 3).

Therapeutic interventions and decision-making

The receipt of the PET/CT report precipitated a crisis in the patient's management and emotional state. The patient and her family interpreted the report as a diagnosis of a second, lethal cancer (pancreatic adenocarcinoma), compounding the devastation of the esophageal cancer diagnosis. Clinically, the distinction was vital. If the lesion were a primary pancreatic cancer, the patient would have two synchronous aggressive malignancies, likely precluding any meaningful treatment. If it were a metastatic lymph node from the esophageal cancer, she had stage IV disease, treated with palliative intent. If it were merely inflammation (false positive), she might still be a candidate for curative surgery.

To resolve this, the multidisciplinary tumor board recommended an EUS with fine-needle aspiration (FNA). This procedure was performed on November 25, 2022. The EUS visualized a large, heterogeneous mass in the celiac axis, encasing the celiac trunk and abutting the pancreas. Transgastric FNA was performed.

Confirmatory Pathology (December 1, 2022)

The cytology and cell block analysis revealed malignant cells. Immunohistochemical staining was performed: the cells were positive for p63 and CK5/6 (markers of squamous differentiation) and negative for CK7, CK20, and neuroendocrine markers. This profile conclusively proved that the "pancreatic mass" was, in fact, a metastatic lymph node from the esophageal squamous cell carcinoma. There was no pancreatic adenocarcinoma.

Based on this result, the final stage was determined to be stage IV (T4 N3 M1). The patient was started on systemic palliative chemotherapy in December 2022. The regimen consisted of a platinum doublet (likely carboplatin/paclitaxel or cisplatin/5-FU), which is the standard of care for metastatic squamous cell carcinoma [1].

Follow-up and clinical outcome

The patient tolerated the initial cycles of chemotherapy with expected side effects. However, follow-up imaging in early 2023 showed limited response to therapy, with stable disease initially followed by progression. The aggressive biology of the tumor, with its significant burden of nodal disease, drove a relentless clinical decline. By mid-2023, the patient had developed worsening dysphagia and cachexia. Supportive care measures, including nutritional support and pain management, were maximized. The patient passed away at home on December 11, 2023, approximately 14 months after her initial diagnosis. The death certificate listed "malignant neoplasm of the esophagus" as the underlying cause of death, with "secondary malignant neoplasm of other sites" (metastasis) as a contributing factor.

Following her death, the family initiated legal proceedings against the imaging center, arguing that the month-long investigation into the "false" pancreatic cancer delayed chemotherapy and deprived the patient of a chance for a better outcome.

## Discussion

This case serves as a paradigm for the complexities inherent in modern oncologic practice, where advanced imaging capabilities often outpace the ability to immediately interpret their clinical significance without invasive confirmation. The discussion of this case requires a multi-faceted approach, examining the technical limits of PET/CT, the clinical impact of the findings, and a rigorous medico-legal analysis of causation.

Diagnostic challenges: specificity and the "false positive" paradox

The core medical issue in this case was the interpretation of the hypermetabolic lesion in the pancreatic bed. To the layperson, a scan showing a "neoplastic mass" in the pancreas implies pancreatic cancer. However, to the oncologist and radiologist, this finding represents a broad differential diagnosis.

Current literature emphasizes that while FDG-PET/CT has a sensitivity exceeding 90% for detecting metastases in esophageal cancer, its specificity is significantly lower, often cited around 65-75% for lymph node involvement [2]. FDG is a nonspecific tracer; it accumulates in any tissue with high glucose transporter (GLUT) expression. This includes not only carcinomas but also macrophages and leukocytes involved in inflammation. More importantly, different types of malignancies look identical on PET. A lymph node completely replaced by metastatic squamous cell carcinoma will have an SUVmax similar to a primary pancreatic ductal adenocarcinoma [2].

In the case presented, the SUVmax of 13.0 was indeed indicative of malignancy. The radiologist's report was technically accurate: the lesion was neoplastic. The error, if one existed, was in the anatomical attribution, perceiving it potentially as a pancreatic origin rather than a peripancreatic lymph node. However, given the lesion's size (3.5 cm) and its distinct separation from the primary esophageal mass, raising the possibility of a synchronous tumor was a prudent radiological safety measure. Ignoring the possibility of a second primary could have led to disastrous consequences, such as performing an esophagectomy on a patient with untreated pancreatic cancer.

Therefore, the recommendation for "clinical and histopathological correlation" was not merely a disclaimer; it was a mandatory step in the standard of care [2]. The National Comprehensive Cancer Network (NCCN) guidelines for esophageal cancer explicitly state that "FDG-PET-positive findings that would alter management (e.g., from curative to palliative) must be confirmed by biopsy" [1]. The medical team's decision to pause and biopsy the lesion via EUS was the only evidence-based course of action available.

Prognostic implications: the biology of celiac metastasis

A central tenet of the plaintiff's claim was that the delay of approximately 30 days to investigate the mass compromised the patient's survival. To address this, one must examine the prognostic significance of celiac lymph node metastasis in esophageal squamous cell carcinoma.

Anatomically, the lymphatic drainage of the distal esophagus flows directly to the celiac axis. Involvement of these nodes (formerly classified as M1a, now often considered regional N3 or M1, depending on the classification system used) represents a breach of the locoregional containment of the tumor [3]. Studies by Chen et al. [3] and Rutegård et al. [6] have demonstrated that the presence of celiac nodal metastasis is one of the single strongest predictors of poor outcome. The median overall survival for patients with celiac metastasis ranges from 8 to 13 months in various cohorts, even with aggressive chemoradiotherapy [3,6].

In this context, the patient's survival of 14 months is actually superior to the historical median for her disease stage. This statistical reality strongly refutes the hypothesis that the outcome was worsened by the diagnostic interval. The disease followed its natural, aggressive biological course. Furthermore, recent population-based data suggest that for advanced cancers, a "time-to-treatment" interval of up to 30-45 days is not associated with inferior survival [4]. In fact, some studies indicate that patients with slightly longer diagnostic intervals often have better outcomes, likely because they undergo more thorough staging that ensures they receive the correct treatment (systemic chemotherapy) rather than incorrect treatment (futile surgery) [4].

Medico-legal analysis: the Simonin criteria

To objectively assess the claim of malpractice, an independent medical expert applied the Simonin criteria, a recognized forensic methodology for establishing the causal link (nexus) between a medical act and alleged damage. The detailed analysis is as follows:

Reality of the Damage (Nature of Harm)

The plaintiff claimed damages for "loss of chance" and wrongful death. The expert identified that the "damage" (death) was real. However, the expert distinguished between the death itself and the cause of the death. The damage was the result of a terminal illness diagnosed in late 2022.

Existence of a Fault (Breach of Duty)

The analysis scrutinized the PET/CT report. Did the radiologists deviate from the standard of care? The expert concluded they did not. The report identified the lesion, characterized it as neoplastic, and advised biopsy. It did not definitively diagnose pancreatic cancer; it raised it as a differential. In radiology, identifying an abnormality and offering a differential diagnosis is the standard of duty. There was no "missed" diagnosis. The fact that the lesion was a metastasis and not a primary tumor is a histological distinction, not a radiological one. Therefore, no medical error (fault) occurred.

Diagnostic Certainty

Subsequent EUS-FNA confirmed that PET/CT correctly identified a malignancy at that location. The scan was a "true positive" for cancer. The legal claim that the report was "wrong" is factually incorrect; the report was right about the presence of cancer, just ambiguous about its cellular origin, which is an inherent limitation of the technology, not an error of the operator.

Temporal Adequacy

The expert reviewed the timeline: PET on November 4, biopsy on November 25, pathology on December 1, and chemo in December. This represents a diagnostic workup completed in under four weeks. In the context of complex oncology cases requiring specialized tests such as EUS, this timeline is within acceptable international standards. There was no administrative negligence or unexplained delay.

Continuity of the Anatomo-Clinical Chain

The expert found a logical progression. The PET findings directly led to the EUS, which directly led to the correct diagnosis of stage IV disease, which directly led to the correct prescription of chemotherapy. The diagnostic path was linear and appropriate. There was no deviation that took the patient off the path of standard care; rather, the "detour" to biopsy the pancreas was a necessary loop to ensure the final diagnosis was precise.

Exclusion of Other Causes

The expert concluded that the patient's death was entirely explained by the pre-existing pathology: stage IV squamous cell carcinoma. This condition has a mortality rate approaching 100% in the short-to-medium term. The influence of the 30-day workup on this outcome is negligible compared to the weight of the tumor biology. The "loss of chance" doctrine requires that a substantial chance of survival was lost; here, the chance of cure was already zero at the time of presentation due to the metastasis.

Communication and the patient experience

While the medical and legal defenses are robust, the case highlights a profound failure in communication. The patient lived for several weeks believing she had two cancers. This psychological trauma is significant. The "expectation gap" refers to the difference between what a patient expects a test to show (a black-and-white answer) and what it actually provides (shades of grey requiring interpretation). To a radiologist, the phrase "consistent with neoplastic nature" is a sterile, technical descriptor. To a patient, it is a death sentence. This case underscores the need for "structured reporting" and better clinician-patient dialogue [5]. The treating oncologist plays a crucial role as the "translator" of imaging reports. It is imperative that clinicians warn patients before a PET scan that "hot spots" are common and often require biopsy, preemptively managing the anxiety that arises from ambiguous results [5]. If the patient had been counseled that "the scan might show spots that need testing, but that does not mean you have a new cancer," the emotional damage, and likely the lawsuit, might have been avoided.

## Conclusions

This medico-legal case report illustrates that correct medical practice does not always align with patient perception. The radiologists and oncologists acted in accordance with the highest standards of care: they identified a suspicious lesion, characterized it with advanced imaging, and rigorously confirmed its nature with biopsy before starting treatment. The alleged "delay" was, in reality, the time required for diligent medical care.

The legal resolution of the case, supported by the forensic application of the Simonin criteria, affirmed that the patient's unfortunate demise was caused by the aggressive nature of esophageal carcinoma, not by diagnostic mismanagement. However, the case serves as a sober reminder to the medical community. While our algorithms and technologies are advanced, our ability to communicate uncertainty remains a work in progress. Improving how we deliver complex, ambiguous news to vulnerable patients is as vital as the accuracy of the diagnosis itself.
